# Seroepidemiological study on the spread of SARS-CoV-2 in populations in especially affected areas in Germany – Study protocol of the CORONA-MONITORING lokal study

**DOI:** 10.25646/7053

**Published:** 2020-11-18

**Authors:** Claudia Santos-Hövener, Markus A. Busch, Carmen Koschollek, Martin Schlaud, Jens Hoebel, Robert Hoffmann, Hendrik Wilking, Sebastian Haller, Jennifer Allen, Jörg Wernitz, Hans Butschalowsky, Tim Kuttig, Silke Stahlberg, Julia Strandmark, Angelika Schaffrath Rosario, Antje Gößwald, Andreas Nitsche, Osamah Hamouda, Christian Drosten, Victor Corman, Lothar H. Wieler, Lars Schaade, Thomas Lampert

**Affiliations:** 1 Robert Koch Institute, Berlin Department of Epidemiology and Health Monitoring; 2 Robert Koch Institute, Berlin Department of Infectious Disease Epidemiology; 3 Robert Koch Institute, Berlin Centre for Biological Threats and Special Pathogens; 4 Charité – Universitätsmedizin Berlin Institute of Virology, National Consultant Laboratory for Coronaviruses; 5 Robert Koch Institute, Berlin Institute Leadership; 6 Robert Koch Institute, Berlin Methodology and Research Infrastructure

**Keywords:** SARS-COV-2, COVID-19, SEROLOGICAL STUDY, CROSS-SECTIONAL STUDY, STUDY PROTOCOL, CORONA HOTSPOT

## Abstract

At a regional and local level, the COVID-19 pandemic has not spread out uniformly and some German municipalities have been particularly affected. The seroepidemiological data from these areas helps estimate the proportion of the population that has been infected with SARS-CoV-2 (seroprevalence), as well as the number of undetected infections and asymptomatic cases.

In four municipalities which were especially affected, 2,000 participants will be tested for an active SARS-CoV-2 infection (oropharyngeal swab) or a past infection (blood specimen IgG antibody test). Participants will also be asked to fill out a short written questionnaire at study centres and complete a follow-up questionnaire either online or by telephone, including information on issues such as possible exposure, susceptability, symptoms and medical history.

The CORONA-MONITORING lokal study will allow to determine the proportion of the population with SARS-CoV-2 antibodies in four particularly affected locations. This study will increase the accuracy of estimates regarding the scope of the epidemic, help determine risk and protective factors for an infection and therefore also identify especially exposed groups and, as such, it will be crucial towards planning of prevention measures.

## 1. Introduction

In December 2019, the first cases of a lung disease caused by a new coronavirus were described in Wuhan, China. Since then, SARS-CoV-2 (Severe Acute Respiratory Syndrome Coronavirus 2) infections and cases of COVID-19 (Coronavirus Disease 2019) have spread worldwide, causing a pandemic with over 12 million confirmed diagnoses and 560,000 deaths. Germany has so far registered 195,000 SARS-CoV-2 infections, causing 9,064 fatalities either of or related to the development of COVID-19 (as at 13 July 2020) [1]. Federal states which are particularly affected with a high cumulative incidence (cases per 100,000 inhabitants) included Bavaria (20 cases per 100,000 inhabitants), Saarland (17.5 cases per 100,000 inhabitants) and Baden-Württemberg (16.6 cases per 100,000 inhabitants). In Germany, the distribution of the COVID-19 pandemic differs widely across all regions and locations. Following a local outbreak caused by a business woman visiting Bavaria from China at the beginning of the year [2], subsequent infections were mainly due to people returning from Italian and Austrian ski resorts. Some municipalities in Germany have registered an overproportionate number of COVID-19 infections. Local infection hotspots have often been related to events where a greater transmission of the virus occurred such as carnival parties, concerts or other festive events [3–7]. Internationally, this phenomenon of indoor events as a possible place of transmission for SARS-CoV-2 infections is familiar [8, 9]. Further relevant situations that drive transmission and are related to living and/or working conditions include crowded shared accommodation or working in the meat industry [10].

The available data on the spread of SARS-CoV-2 infections in Germany has so far been based on confirmed SARS-CoV-2 case numbers reported to the local health authorities in line with Germany’s Protection against Infection Act (IfSG). These cases are diagnosed by polymerase chain reaction (PCR) tests. Based on current scientific data, however, an unknown proportion of SARS-CoV-2 infections can be assumed to take an asymptomatic or mild course, which means that many subclinical or mild infection courses are not diagnosed as SARS-CoV-2 infections. Furthermore, in line with the recommendations from the Robert Koch Institute (RKI), the European Centre for Disease Prevention and Control (ECDC) and the World Health Organization (WHO), PCR tests are conducted only with symptomatic patients likely to test positive for SARS-CoV-2. So currently, reported case numbers do not provide a reliable estimate of the actual prevalence of past SARS-CoV-2 infections in the population. In the COVID-19 Case-Cluster-Study, which was conducted in the Gangelt municipality (North Rhein-Westphalia) between March and April 2020, researchers reported an IgG seroprevalence of 15.5% and a factor of five for undetected infections (with regard to the number of registered SARS-CoV-2 cases) [11]. However, due to a different method the comparability of results is limited.

Due to the high number of cases, seroepidemiological data from particularly affected locations facilitates a rather accurate estimate of past infections and provides a good indication of the number of undetected SARS-CoV-2 infections. Furthermore, research into the risk and protective factors for an infection help identify high risk groups, and this is crucial in terms of putting prevention measures in place. Conclusions related to the burden of disease, the number of asymptomatic infections, as well as the dynamic of infections can be drawn and, in part, transferred to locations not as impacted by the epidemic.

In the context of the CORONA-MONITORING lokal study, antibody prevalence and the proportion of active infections was determined in four municipalities particularly affected by SARS-CoV-2 and a cumulative incidence of over 500 registered cases per 100,000 inhabitants over a timespan of one month.

At each survey location the study aims to:

1. Determine seroprevalence (proportion of the population that has been in contact with the virus) for each survey location by age group and sex,
2. Calculate the proportion of undetected infections,
3. Identify the proportion of asymptomatic infections.

Due to the limited number of cases, some analyses cannot be conducted for each location; but will be conducted with aggregated data of all four sample points:

1. Sensitivity analyses of infection rates and the proportion of undetected infections by taking into account the data of non-responders, as well as the data on reported and/or deceased cases.
2. Presenting infection rates differentiated by exposure contexts (living conditions including household size, having children to care for where applicable; contact intensity in the work environment (during the pandemic) and use of public transport).
3. Calculation of the proportion of undetected cases relative to the number of reported cases differentiated by risk group status (age group 65 years and older, pre-existing conditions) and age group, sex and education.
4. Differentiation of symptomatic infections by sex and/or age group, as well as by exposure contexts (living conditions including household size, having children to care for where applicable; contact intensity in the work environment (during the pandemic) and use of public transport).
5. Identification of risk and protective factors for a SARS-CoV-2 infection and the extent to which it is embedded in the respondent’s living, family and occupational situation.
6. Calculation of infection mortality (where case numbers were sufficiently high, stratification by age and sex).

Furthermore, the study aims to create a basis for longitudinal studies that will potentially facilitate follow-ups on the possible sequelae of people who have experienced a SARS-CoV-2 infection.

## 2. Methodology

### 2.1 Study design and sampling

#### Study design

The CORONA-MONITORING lokal study is a population-based, seroepidemiological observation study that will conduct cross-sectional examinations at four locations in Germany which were particularly affected by the COVID-19 epidemic. An ongoing acute transmission of SARS-CoV-2 would call for a repetition of cross-sectional surveys or supplementing the results with longitudinal examinations with serial serological testing and interviews with a selected group of participants.

#### Sample

The study will be conducted at four municipalities particularly affected by the COVID-19 epidemic (defined as a reported cumulative SARS-CoV-2 incidence of over 500 cases per 100,000 inhabitants one month before the beginning of data collection) among 2,000 individuals for each study. Municipalities are selected with regard to the epidemiological developments shortly before the local start of the study. The selection criteria for municipalities are a past or ongoing transmission as well as the willingness of local authorities to contribute towards the study.

A random sample from population registries is provided to the RKI. This includes adults aged 18 years and older with no upper age limit who are registered residents in one of the surveyed municipalities. Proportional sampling is applied, i.e. population registries are asked to provide 4,000 to 5,000 randomly drawn addresses not stratified by sex and/or age group. Based on an expected response rate of between 60% and 70%, at first 2,900 randomly selected individuals are then contacted in writing. If the response rate is lower than expected, further random samples will be drawn within sex and/or age groups underrepresented among participants from the first sample. When further survey waves are conducted, this step is repeated. The municipalities that have been selected so far are Kupferzell (Baden-Wuerttemberg), Bad Feilnbach (Bavaria) and Straubing (Bavaria).

#### Inclusion and exclusion criteria

Individuals will be included

▶ if they are 18 years or older,
▶ are registered residents of one of the four study municipalities,
▶ can provide written consent to participate in the study, or, where necessary, written consent is provided by a legal representative,
▶ are able to take part in the interviews (where necessary with the help of relatives) and the examinations at the study centres or during home visits.

People who lack the necessary German language skills or where it is unclear whether they understand the study information leaflets or the consent forms are excluded from participating. Currently, we are considering translating the survey material into English and further locally relevant languages and seeking the services of interpreters.

### 2.2 Study implementation

#### Recruiting and non-response

**Figure 1:**
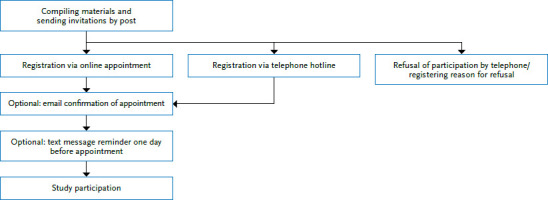
CORONA-MONITORING lokal study recruitment procedure Source: Own depiction

The individuals selected randomly from the population registries of the corresponding municipalities are first invited in writing to take part in the study. They receive an invitation by mail with materials informing them about the study (study information, data protection declaration, consent form and a personalised ‘participation schedule’). Those who are willing to participate can make an appointment at the study centres choosing from the days scheduled for the study either via an online calendar or by phone at the study hotline (Figure 1). Depending on the response rate, a reminder is sent to people who have been invited but neither responded nor made an appointment after about one week.

People in the age group 60 years and older, who have been defined as a high risk group for a COVID-19 infection, receive an adapted invitation letter that explicitly offers the option of a home visit [12]. A telephone hotline with specially trained staff has been established to take into account the individual needs and requests of elder and elderly people in the run-up to home visits. People aged under 60 years with limited mobility or who fear an infection and therefore cannot or do not want to go to a study centre, are offered home visits upon request as a measure to limit the effects of selection bias.

People who contact survey staff and refuse to take part are asked to state the reasons for their decision. People who do not react to the letter and with whom no contact is therefore established will be chased-down after the field work has been completed and will be asked to fill out a non-responder questionnaire to estimate the representativeness of the study sample.

#### Study centres

Per municipality, one to two temporary study centres will be established. Centres will consist of a bus for examinations and a rented space. The latter will count with one to two receptions and two examination rooms as well as a laboratory (with a centrifuge and a laptop). The bus for examinations provides two further examination rooms.

#### Study staff

The study staff includes physicians, staff who have completed training in a medical profession (health care and nursing, geriatric care, medical assistants), nutritionists and administrative employees. All in all, the study teams consist of around 25 individuals.

In preparation for the CORONA-MONITORING lokal study the following training sessions were organised:

▶ overview of the study objectives
▶ overview of SARS-CoV-2 and COVID-19
▶ measures of infection protection (including disinfecting measures)
▶ working with personal protective equipment
▶ participant reception and informed consent
▶ conducting home visits
▶ taking blood specimens and oropharyngeal swabs
▶ specimen processing, storage and transport
▶ break periods

All the staff involved in the study took part in these training sessions (training in taking blood samples and conducting oropharyngeal swabs only for the corresponding staff).

#### Study procedure

**Figure 2:**
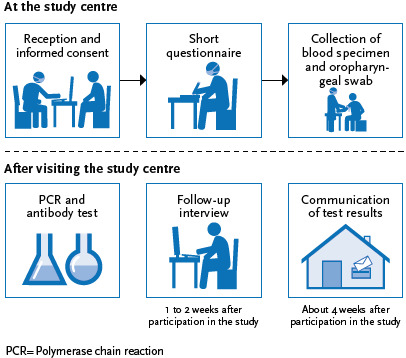
CORONA-MONITORING lokal study procedure Source: Own depiction

Figure 2 shows the study procedure. Upon arrival at the study centre, participants identify themselves with a valid identity document. Reception staff then informs them about the objectives and purpose of the study (as well as the fact that participation is voluntary) and answers any questions that arise. Together with the invitation, participants receive a consent form, but are asked to sign it only once they arrive at the study centre.

Participants then fill out a short questionnaire, provide a blood sample and an oropharyngeal swab. Oropharyngeal swabs are taken out of the opened mouth using the Copan Group (Brescia, Italy) Copan Liquid Amies Elution Swab transport system. For venous blood collection, the Vacutainer System 8.5ml tube BD Vacutainer SST II Advance (Fa. Becton Dickinson GmbH, Heidelberg) with separating gel and clotting activator is used.

Blood samples and oropharyngeal swabs are immediately taken to the laboratory. The swabs are stored in a fridge and blood samples centrifuged for 12 minutes at 4.400 RPM (corresponding to 3000 x g) 30 to 45 minutes after being taken in an Eppendorf model 5702 lab bench centrifuge (Eppendorf AG, Hamburg) and filled into two serum tubes (screw caps 10 ml PP sterile, Sarstedt Ag & Co. KG, Rheinbach) and then also stored in the fridge. Consent forms and written questionnaires are sent with the specimens collected and stored at 4°C in actively cooled boxes (Dometic Group CoolFreeze CF 35; Dometic Germany GmbH, Elmsdetten) with a daily shuttle to the RKI for further processing.

Home visits are conducted by teams of two people: one person to take specimens and one person as a driver. Procedures during home visits match those at the study centres. After the home visit, the driver takes the consent form, the questionnaire and the specimens to the study centre for further processing.

Around one to two weeks after the examination, a follow-up interview via an online questionnaire is scheduled. If a participant prefers, this interview can also be conducted as a telephone interview (Table 1).

#### Infection prevention during data collection

At the study centres and during home visits, participants are asked to wear facemasks and are, if necessary, provided with one. Participants are also asked to maintain physical distance. During specimen collection, the examination staff wear personal protective equipment and particle filtering FFP2 masks. Examination rooms are regularly cleaned, aired and surfaces disinfected after every examination. Participants with cold symptoms are asked not to come to the study centre and whenever possible an appointment for a home visit is made.

#### Communication of results

Participants receive their PCR laboratory and antibody test results in the form of a pseudonymised result report. The following values are tabulated with their corresponding reference intervals:

▶ PCR test result for SARS-CoV-2 virus material on the oropharyngeal swab (positive/negative).
▶ ELISA (enzyme-linked immunosorbent assay) for SARS-CoV-2 IgG antibodies in the blood (positive/marginal positive/negative).

Before communicating the results, a trained study physician conducts a plausibility check for the test results. The administrative survey staff send out the pseudonymised result report accompanied by a personalised letter. Notifiable laboratory results are forwarded to the responsible health office (in writing) within 24 hours and the participant is informed about the result by the medical study staff (by telephone and in writing). In addition, infected participants receive the RKI’s information leaflet on self-isolation.

#### Quality assurance (QA)

Quality assurance measures during the study procedures are organised through an internal QA taskforce, which has previously been established at the RKI for other studies.

**Info box:**

**Sensitivity, specificity and cross-reactivity**

**Sensitivity** indicates how well a test can correctly identify a person with SARS-CoV-2 specific antibodies.

**Specificity** indicates how well a test can correctly identify a person without SARS-CoV-2 antibodies.

**Cross-reactivity** describes the capacity of antibodies to bind to antigens with similar docking sites. With regard to SARS-CoV-2, this means that an antibody does not bind only to SARS-CoV-2 but potentially also to other coronaviruses spread in Germany (such as HCoV-OC43, HCoV-HKUl).

At all stages of data collection, Standard Operating Procedures (SOP) have been defined. The study team receives training based on a fixed training protocol before data collection begins. Furthermore, before actual data collection, a pretest was run to test and where necessary adapt all steps. Continuous supervision by the leading field staff and the QA team during data collection is supplemented by regular further training provided to the study team; these training sessions are firmly anchored within the schedule. Where necessary, additional individual training sessions can be carried out.

All the steps during laboratory examinations are documented (from blood sample collection to arrival of the specimens at the RKI’s central epidemiological laboratory) to accomplish QA and to make sure that samples are not mixed up.

### 2.3 Survey methods and content

#### Laboratory diagnostics

Two in-house PCR tests are used to test for the SARS-CoV-2 genome. Test 1 detects the E-gene adapted to [13] and is monitored with a simultaneous PCR to offset possible errors with ribonucleic acid (RNA) extraction as well as a possible PCR inhibition. Test 2 is specific to SARS-CoV-2, is located in the ORF1ab region and can confirm not only the presence of the SARS-CoV-2 genome but also cellular nucleic acids and therefore successful sample extraction. Under the test conditions applied, both SARS-CoV-2 tests have a limit of detection of <10 genomes/reaction and therefore, at the analytical level, have a sensitivity and specificity (Info box) of nearly 100%. Due to the relatively short timeframe during which the virus is detectable in patients’ throats and difficulties with conducting swabs, the actual sensitivity of the test is below 100%.

To detect IgG antibodies against the new coronavirus, the study uses Euroimmun’s (Euroimmun Medizinische Labordiagnostika AG, Lübeck) commercial laboratory test ‘Anti-SARS-CoV-2-ELISA (IgG)’. The test has been validated by a number of laboratories (for example by the National Consultant Laboratory for Coronaviruses at the Charité-Universitätsmedizin Berlin, Professor Christian Drosten). With a sensitivity of 93.8% and a specificity of 99.6%, the test is of high quality and of low cross-reactivity (Info box). The use of this test in numerous further national and international studies makes comparisons of seroprevalence easier. The analyses are automated using Euroimmun’s high throughput ‘EUROLab Workstation ELISA analyser.

As in all test procedures with a specificity of <100% – in particular when seroprevalence in the population is low – a certain proportion of false positives is inevitable. A neutralisation test was therefore performed on all ELISA reactive samples at the National Consultant Laboratory for Coronaviruses at the Charité-Universitätsmedizin Berlin. The result of this test was used for scientific evaluation. The exact method of this plaque reduction neutralisation test is described here [14].

IgG antibody tests indicate whether someone has in the past had contact with the new coronavirus. Whether, and if yes, for how long an individual is then immune to the new coronavirus, cannot yet be safely said. Even people with a positive antibody test should therefore keep to the recommended hygiene and behaviour rules in their private and professional contexts. The same in any case applies to people without a positive antibody test.

#### Interviews

Laboratory examinations to detect an acute or past SARS-CoV-2 infection are supplemented by interviews with study participants to gain further information on a set of questions such as potential exposure, susceptibility, symptoms and medical history. Interviews consist of two parts: a short questionnaire that participants fill out either at the study centre or during home visits in the form of a written questionnaire, as well as a more detailed questionnaire that participants are asked to fill out one to two weeks after the appointment. Preferably, the administration is web-based, which means that participants receive access to an online questionnaire which they are asked to complete. Participants that cannot or do not want to fill out this questionnaire online can complete it via a computer-assisted telephone interview (CATI). Table 1 summarises the content of both questionnaires. As far as possible, established survey instruments and validated scales are used [15–20], and these are complemented or modified to the specific survey mode and focus of the study. The questionnaire is adapted to reflect specific events that have taken place at each survey location and the periods of time people are asked to remember regarding the epidemio-logically-relevant events at a specific location. Following data collection, all non-responders receive a non-responder questionnaire to gain an understanding of their reasons for not taking part.

## 3. Expected results

We now present the expected results and the statistical procedures involved in data analysis.

###  

#### Statistical analysis and estimation of sample size

Data analysis involved applying a weighting variable to better reflect the structure of the general population in the selected municipalities regarding age and sex. In addition, weighting is applied regarding education by sex and age group. The marginal distribution is approximated referring to the administrative district based on micro census data 2017 [21].

Weighting allows true but unknown parameters in the population to be better reflected although it is also related to an increase in the variance of estimators gained in the study. Based on experiences made in previous studies and assuming a relatively high willingness to participate and low selectivity, we set this factorto estimate case numbers at 1.43 (=1/(70%), where 70% describes the ‘effectiveness’ of the weighting variable, a measure for the spread of weights). For younger adults (18-to 34-years-old) and the older age group (65 years and older) based on previous studies effectiveness is set slightly lower (at 60% and 65%, respectively) [22, 23].

Based on these assumptions, with a net sample of n=2,ooo individuals we can achieve the accuracies given in Table 2 for the estimates of seroprevalence for the general population of the corresponding study location, as well as for the population stratified by age group. Estimates are presented for an estimated seroprevalence of 3%, 5%, 10% and 15% in the general population with a distribution among age groups that corresponds to the distribution of reported cases in regions with a high number of infections (own calculation based on population registry data; as at end of March 2020).

If the requirement for estimate precision defines that the variation coefficient of the prevalence estimate is to be less than one sixth (which is equivalent to requiring that the bottom limit of the confidence interval should be at least two thirds of prevalence), then all the expected confidence intervals are in this sense acceptable for estimates regarding the general population. For the estimates stratified by age group, this requirement is not met if seroprevalence is only 3%, or only 3% or 5% for the oldest age group. However, it is possible to increase the precision of the estimators for individual age groups, if we group estimators for several particularly affected municipalities. Moreover, 3% tends to be the lower limit for the seroprevalences that can be expected in particularly affected locations.

#### Descriptive analysis

Descriptive analyses are first conducted for the following parameters:

▶ description of response and non-response,
▶ sociodemographic characteristics (at first age and sex; in analyses with follow-up interviews socioeconomic status, household composition, etc.),
▶ prevalence of SARS-CoV-2 IgG antibodies in blood samples, and/or positive oropharyngeal swab test in the corresponding population (at first stratified by age and sex, later by socioeconomic status and other relevant indicators),
▶ prevalence of symptomatic and asymptomatic cases (based on test history),
▶ undetected case rate of antibody prevalence relative to the number of reported cases,
▶ antibody prevalence relative to local events visited.

#### Further and multivariate analyses

Further analyses look at the following questions:

▶ Understanding SARS-CoV-2 risk and protective factors, as well as the extent to which they are embedded in the participants’ living, family and occupational situation.
▶ Sensitivity analysis of infection rates and the proportion of undetected cases that take into account non-responder data as well as data on reported cases and fatalities.
▶ Estimating the proportion of undetected cases for reported cases stratified by risk group yes/no (age group 65 years and older, other conditions) and age group, sex, and education.
▶ Influencing factors and social contacts, mental health and everyday life.
▶ Asymptomatic infections stratified by sex and/or age group, as well as by exposure contexts (living conditions including household size, having children to care for where applicable; contact intensity in the work environment (during the pandemic) and use of public transport).
▶ Infection fatality stratified by age and sex.

## 4. Discussion

Studies in locations particularly affected by SARS-CoV-2 can contribute towards a better understanding of the spread of the infection. The proportion of people who have actually had an infection can help to estimate the number of undetected cases relative to the number of reported cases. This will provide a better idea of the local extent of the epidemic as well as the proportion of asymptomatic infections, and, by identifying active infections, the current infection dynamics.

Currently, a number of seroepidemiological studies on SARS-CoV-2 are being conducted in municipalities particularly affected [24, 25]. In the context of the COVID-19 Case-Cluster Study, which was conducted during March and April in Gangelt, a municipality particularly affected by SARS-CoV-2 in the Heinsberg district (North Rhein-Westphalia), an IgG-seroprevalence of 13.60% was described. After monitoring sensitivity (90.9%) and specificity (99.1%), seroprevalencewas corrected to 14.11% [11]. Neutralisation tests were conducted but were not considered for the definition of antibody positive samples. When including acute confirmed infections as well as self-reported PCR-positive results the proportion of persons infected was described as 15.53%. A factor of five was estimated for the number of undetected cases. However, the methodology was not completely comparable with that of the CORONA-MONITORING lokal study, in particular because in our study only samples with a positive neutralisation test result were considered as confirmed positives.

The focused analysis of locations particularly affected with high rates of infection is an important contribution to understanding infection dynamics and the developments of population immunity while taking into account a diverse range of transmission dynamics. For example, the study can provide information on the likelihood of transmission, as well as on risk factors for a severe course of the disease, which are relevant not only to the local development of infections. Additional longitudinal studies with repeated serological testing of population subsamples can, due to the expected greater number of cases, provide information on the development of the epidemic and immunity of the population overtime. Furthermore, we can also keep track of people who have tested positive for SARS-CoV-2 antibodies, which should provide findings on the long-term effects of COVID-19. However, as these studies are focused on local hotspots and population structures, the results cannot be transferred to the general population and studies in particularly affected municipalities therefore cannot indicate the prevalence of SARS-CoV-2 antibodies in the general population in Germany. Possible limitations of the CORONA-MONITORING lokal study are a potential selection bias (only German speakers) which hopefully can be countered in the future by providing materials in several languages and interpreters. Recall bias is also a potential issue because many questions refer to the long time period since the beginning of the COVID-19 epidemic in Germany. A further point of discussion relates to how long SARS-CoV-2 antibodies remain detectable, as some studies indicate a decrease of antibody titers overtime [26]. Still, sero-surveys in particularly affected municipalities remain important to understand the dynamics of the COVID-19 pandemic. At the Robert Koch Institute, along with the CORONA-MONITORING lokal study, three further studies to determine seroprevalence are being conducted to achieve a more complete picture of infection dynamics. In addition to serological analyses of blood donor samples for SARS-CoV-2 antibodies (SeBluCo study) [27] that have in preliminary findings determined an antibody prevalence of 1.3%, a Germany-wide study is being planned to better estimate seroprevalence in the general population. A further study is being conducted to accompany the re-opening of childcare facilities that will provide answers on the role of children (aged 1 to 6) in the transmission of the disease [28].

In the context of the CORONA-MONITORING lokal study, SARS-CoV-2 seroprevalence is determined for four particularly affected locations. Findings can contribute to a better understanding of the local infection dynamics, but also allow comparisons of the local situations. A broader analysis of the data will allow us to determine risk and protective factors for an infection and a severe disease course and therefore identify high risk and exposure groups, which is essential for the planning of prevention measures.

## Key statements

- The spread of SARS-CoV-2 infections differs widely across Germany.
- Local infection hotspots can apparently often be traced to certain events or situations where pathogen transmission is increased.
- As part of the study, 2,000 participants per location are examined in a temporary study centre or during home visits for an active SARS-CoV-2 infection as well as SARS-CoV-2 IgG antibodies.
- Seroprevalence figures allow an estimate of the number of undetected and asymptomatic SARS-CoV-2 infections.
- The CORONA-MONITORING lokal study surveys the population antibody status at four locations in Germany that have been particularly affected by the SARS-CoV-2 pandemic, and this will enable an estimate of the local population seroprevalence.

## Figures and Tables

**Table 1:** CORONA-MONITORING lokal study, content of the questionnaires Source: Own depiction

**Short questionnaire** **(SAQ-P provided at the study centre or during home visits)**
Age and sex Respiratory symptoms COVID-19 diagnosis and care Quarantine and reasons for quarantine Travel anamnesis Compliance with physical distancing Taking part in events General health Medical history Recent hospitalisations Smoking Work situation (for example contact with patients) Education, employment status, household
**Detailed questionnaire** **(follow-up questionnaire as SAQ-W or CATI)**
Age and sex Diseases, medications, vaccinations Testing and medical diagnosis of COVID-19 COVID-19 symptoms Health care (for example not utilising healthcare services) Health behaviour (for example physical activity or alcohol consumption) Mental and psychosocial health (for example depression) Social relations and contacts Everyday life and leisure time (for example compliance with hygiene and physical distancing rules, wearing facemasks) Recent events in the region Working conditions Living situation Mobility Travel Health at higher age (60 years and older) Acceptance of containment measures Risk perception Detailed sociodemography

SAQ-P = self-administered questionnaire on paper

SAQ-W=web based self-administered questionnaire

CATI=computer-assisted telephone interview

**Table 2:** Expected accuracy of estimators for the (sero)prevalence of SARS-CoV-2 infections in the population aged 18 years and older in particularly affected locations in Germany Source: Own depiction

Population	Age group	Seroprevalence	Expected width of the 95% confidence interval
	years	%	%
General population	Total	3 5 10 15	2.2–4.0 4.0–6.3 8.5–11.7 13.2–17.0
Population by age group (expected seroprevalence: 3%)	18–34 35–49 50–64 ≥65	3.7 4.0 4.1 2.3	2.2–6.3 2.4–6.6 2.5–6.9 1.1–4.8
Population by age group (expected seroprevalence: 5%)	18–34 35–49 50–64 ≥65	6.2 6.7 6.9 3.8	4.1–9.2 4.5–9.8 4.6–10.2 2.2–6.8
Population by age group (expected seroprevalence: 10%)	18-34 35-49 50-64 ≥65	12.4 13.4 13.8 **7.7**	9.4–16.3 10.2–17.3 10.5–18.0 5.1–11.4
Population by age group (expected seroprevalence: 15%)	18-34 35-49 50-64 ≥65	18.6 20.1 20.7 11.5	14.9–23.0 16.2–24.5 16.7–25.4 8.3–15.8
